# Case Series: The use of Lithium in Bipolar Affective Disorder and End-Stage Renal Disease

**DOI:** 10.1192/j.eurpsy.2024.911

**Published:** 2024-08-27

**Authors:** K. Corrigan, D. Larkin, G. Giusti, M. Gallagher, A. Guerandel

**Affiliations:** ^1^St Vincent’s University Hospital, Dublin, Ireland; ^2^The University of Pisa, Pisa, Italy

## Abstract

**Introduction:**

Lithium is a highly effective treatment in the management of Bipolar Affective Disorder (BPAD) however it is associated with increased risk of developing chronic kidney disease. There is a lack of clear guidance on alternative approaches to managing those individuals that require cessation of lithium due to progression to end stage renal disease (ESRD).

**Objectives:**

We discuss two patients with BPAD on lithium therapy who have developed ESRD. In both cases, lithium was discontinued due to ESRD, with alternatives trialled. In one case, the patient continues to be managed without lithium, whereas in the second, a decision was made to recommence lithium at a low dose. We reviewed the literature to provide meaningful context to the cases.

**Methods:**

Case 1 This patient with a long history of BPAD and multiple medical co-morbidities experienced progressive decline in renal function. A decision was made to cease lithium therapy with close monitoring for signs of affective relapse. The patient was stabilised using a combination of sodium valproate and quetiapine. Since cessation of lithium, the patient has required a significant increase in support from the CMHT and more frequent admissions to manage mood and anxiety symptoms that cause significant subjective distress.

**Results:**

Case 2 This patient had a long history of stable BPAD, with no episodes of illness for over 30 years. Unfortunately they developed CKD and despite a significant reduction in lithium over time, they developed ESRD requiring haemodialysis. Lithium was discontinued leading to a manic relapse of BPAD requiring a prolonged admission and a combination of carbamezapine, olanzapine, escitalopram and clonazepam to stabilise their mental state. Following discharge home, their mental state failed to reach baseline and they reported significant anxiety symptoms and memory impairment. Following protracted assessment and support they were deemed unfit for renal transplant and a decision was then made by the patient, their family, nephrology and psychiatry to recommence lithium therapy whilst on haemodialysis. Their anxiety and functioning improved significantly following the reintroduction of low dose lithium, allowing the withdrawal of other neuroleptics.

**Conclusions:**

Both cases required an individual approach to balance physical and mental health considerations. There are no clear markers to predict if a patient will respond to alternative mood stabilisers, nor is there a guarantee that kidney function will improve or stop declining when lithium is discontinued. Decisions should reflect patient preference and balance risks associated with relapse and of declining ESRD.

**Disclosure of Interest:**

None Declared

